# THE EFFECTS OF EARLY-LIFE ADVERSITY ON WOMEN’S COGNITIVE HEALTH AFTER AGE 50

**DOI:** 10.1093/geroni/igad104.3638

**Published:** 2023-12-21

**Authors:** Kimson Johnson

**Affiliations:** University of Michigan, Ann Arbor, Michigan, United States

## Abstract

According to the Alzheimer’s Association, 6 million people age 65 and older are living with Alzheimer’s Disease in the United States, and nearly 4 million are women. Few research studies have examined the impact of adverse childhood events (ACEs) on cognitive health among women over 50. Evaluating gender-specific differences in predictors of cognitive health can help clarify mechanisms that hinder or magnify gender-based health differences and provide information on ways to narrow gender disparities in dementia. The study examined the relationship between ACEs, cognitive impairment, and incident dementia using data from the Health and Retirement Study Cross-wave 2015-2017 Life History Mail Survey (N = 4,980; Mage = 67.6). Cognitive status was measured using the 2016 Langa-Weir three category indicator: Normal, Cognitive Impairment but No Dementia (CIND), and Dementia. Compared to women exposed to 0 ACEs, women exposed to 1 ACE had a higher relative risk for CIND and dementia (RRR=1.71, p<.05; RRR=2.44, p<.05). Women exposed to 3 ACEs had a higher relative risk for dementia (RRR=3.43, p<.05). Non-Hispanic Black women exposed to 3 ACEs had a lower relative risk for dementia (RRR=0.18, p<.05) than white women. Women with 12 years of education have a higher relative risk for CIND and dementia (RRR=2.27, p<.05; RRR=3.45, p<.05) than women with >13 years of education. Although women’s life expectancy has increased over the past few decades, women remain at risk for delayed identification of cognitive decline. Future research should examine how other critical social determinants of health influence women’s cognitive health after 50.

